# Environmental Preferences and Concerns of Recreational Trail Runners

**DOI:** 10.3390/ijerph21010097

**Published:** 2024-01-16

**Authors:** Leah Rosenkrantz, Nadine Schuurman, Scott A. Lear

**Affiliations:** 1 Faculty of Environment, Simon Fraser University, 8888 University Drive, Burnaby, BC V5A 1S6, Canada; 2 Faculty of Health Sciences, Simon Fraser University, 8888 University Drive, Burnaby, BC V5A 1S6, Canada

**Keywords:** online survey, runnability, outdoor environments

## Abstract

Trail running is a fast-growing sport, linked to improvements in both physical and psychological well-being. Despite its popularity, the preferences of trail runners are not well known. The objective of this study was to examine the environmental preferences and concerns of trail runners with respect to age and gender. We conducted a cross-sectional survey of recreational trail runners. A total of 548 people responded, of which 50.1% of respondents were women and 44.2% were men. The sample was distributed relatively evenly across age groups, up to 54 years; respondents over 55 represented only 9.4% of the sample. Comparisons of runner characteristics by gender indicated significant differences (*p* < 0.05) according to age, distance run per week, and number of days run per week. Certain runner preferences also differed significantly by gender, including importance of running around others, the type of trail races they seek, and whether or not they like to seek “vert” or elevation in their runs. Major concerns for both genders while running included lack of cell reception (Men: 33.8%; Women: 50.8%) and getting lost (Men: 26.8%; Women: 35.5%). Comparisons of the results of this study help to strengthen our understanding of trail runners’ environmental preferences and concerns and can be used to guide future design and maintenance of trail environments to encourage greater participation in the sport.

## 1. Introduction

Trail running, a sport and leisure activity that combines running and hiking, has been growing at a global rate of 12% yearly for the last decade and has recently been recognized by the International Association of Athletics Federation as a new running discipline [1,2]. Already popular in Europe and North America, the sport is now rapidly expanding into Asia and South America [3]. It comprises various natural off-pavement terrains, including forest paths, dirt roads, and alpine trails. Trail running, like road running, can help improve physical and psychological well-being [4,5,6,7,8,9]. Studies have found that running reduces the risk of hypertension, Type 2 diabetes, osteoarthritis, and respiratory infection and, even in low doses, is associated with a considerable reduction in cardiovascular and all-cause mortality [10,11,12,13]. The mental health benefits are numerous, including a reduced risk of depression, anxiety, and overall stress [7]. Trail running, in particular, has the added health benefit of being immersed in nature, which is known to have its own wide-ranging health benefits, including significant reductions in blood pressure, cortisol levels, incidence of diabetes, and all-cause mortality, to name but a few [14,15]. In addition, trail running often includes more vertical gain compared to road running, offering the possibility for trail runners to improve their cardiovascular fitness and strengthen different muscles as they run up hills or mountainsides.

Despite its growing popularity and health benefits, the preferences of runners, specifically trail runners—a diverse group with varying backgrounds and motivations—are not well known [16,17]. The composition of outdoor spaces influences where and how people run, yet environments that positively influence participation in the sport are scarcely studied [8]. The lack of studies on conducive running environments is surprising, given the numerous health benefits of running and consensus on the impact of the built environment on mobility.

Most of the literature focuses on environmental characteristics that improve walkability and cycling, excluding the specific needs of runners [18,19]. Except for a handful of studies, geographers have engaged little with running scholarship [5,15,18,20,21]. Extending geographic paradigms to running research holds great potential, given the necessity to understand the space where this popular activity takes place and the opportunity to modify the built environment to promote the adoption of the sport and its many health benefits. Preferences for environments are likely to differ significantly among trail and road runners, recreational and competitive runners, as well as commuting and leisure runners, due to varying aims and motivations [16,22,23]. As such, research is needed on the varying preferences within the trail running community to better design trails and improve their safety for the runners that use them. Features like improved signage or increased cell reception in a trailed area may be what encourages new runners to try the sport or assuage the concerns of experienced runners looking to expand their routes.

The objective of this study is to examine the type of built and natural environments trail runners prefer with respect to age and gender and the safety and health concerns that may affect trail runners’ choice of environment. Results are examined in the context of findings from our previous study on recreational road runners [23]. Our aim is to deepen the understanding of what constitutes a positive trail running environment, to advance and inform future planning decisions, and to create more places for individuals to pursue this health-promoting activity.

## 2. Materials and Methods

This study was based on a cross-sectional survey. Methods for this study closely follow that for a study on road running, with survey development and recruitment following the same protocols [23]. A summary of methods specific to this study, including study population and design are summarized below.

### 2.1. Study Population

The study population consisted of a sample of people who self-identify as trail runners. No constraints were established concerning the gender, ethnicity, country of residence, or ability of the respondent; however, for ethics purposes, we limited the survey to only those aged 19 years and older. An a priori power analysis (G*Power) was conducted to estimate the minimum sample size required to conduct the analysis. The power analysis suggested a chi-square test of independence (df = 6) using a medium effect size, α = 0.05, and 95% power suggested a minimum sample size of n = 232. We recruited trail runners through advertisements placed in North American running magazines and running-specific websites, as well as targeted social media ads on Facebook and Instagram. We also recruited through local run clubs and running apparel stores with postcard-style handouts. We incentivized participation in the survey with the chance to win one of three CDN 300 gift cards from a running store.

### 2.2. Study Design

Trail runners who were interested in participating in the study were directed to fill out the survey, hosted on the SurveyMonkey online platform. Those who self-identified primarily as a trail runner were directed to fill out 10 questions, which included a variety of Likert scale and multiple-choice questions (Table A1). Questions fell into one of three categories: the respondents’ running profile (including age, gender, and running routines), their environmental preferences for running, and their environmental concerns for running. Written informed consent was sought prior to participation in the survey. Approval for this study (ethics approval #2019s0322) was granted by Simon Fraser University’s Research Ethics Board.

### 2.3. Data Analysis

Descriptive analysis was conducted on all survey answers using Excel, V.16.45. Chi-square tests of independence were performed using SPSS, V.27 to assess whether meaningful differences between genders existed in respondent’s answers to survey questions. Missing responses and respondents who identified as other or did not identify their gender were excluded from this analysis.

Chi-square tests of independence require a minimum expected cell count and independence of responses. For these reasons, respectively, chi-square tests could neither be conducted to assess the presence of meaningful differences across age groups by question (due to not meeting the minimum cell count requirement) nor for the one question permitting more than one response (due to lack of independence of responses). As such, we only present these results descriptively.

NVivo, version 12.6.0 was used to conduct thematic and content analysis on questions that allowed free text responses when “other” was selected. Responses to free text were coded by gender to determine whether common themes diverged between men and women.

## 3. Results

Five-hundred and forty-eight trail runners responded to the survey. Respondents were included in the analysis if they answered at least one question in the survey. Response rates varied by question, ranging from 88.7% to 92.2%.

### 3.1. Trail Runner Profile

Two-hundred and sixty-two (50.1%) respondents identified as a woman, 231 respondents (44.2%) identified as a man, and one respondent (0.2%) identified as another gender; 29 respondents (5.5%) did not answer this question. The sample was distributed relatively evenly across age groups, up to 54 years (Table 1) (Table A2).

On average, respondents had been running for 11.43 years (range = 0.5–55 years). Over half of respondents (59.2%) had been running for 10 years or less. For women, this average was 11.1 years (range: 1–55 years); for men, it was 11.9 years (range: 0.5–50 years), but the difference between gender was not statistically significant. The person who identified as another gender had been running for less than five years. With the increasing age category, the years of running also increased from an average of 5.7 years in those 19–24 up to 26.9 years for those ≥ 65 years.

**Figure 1:**
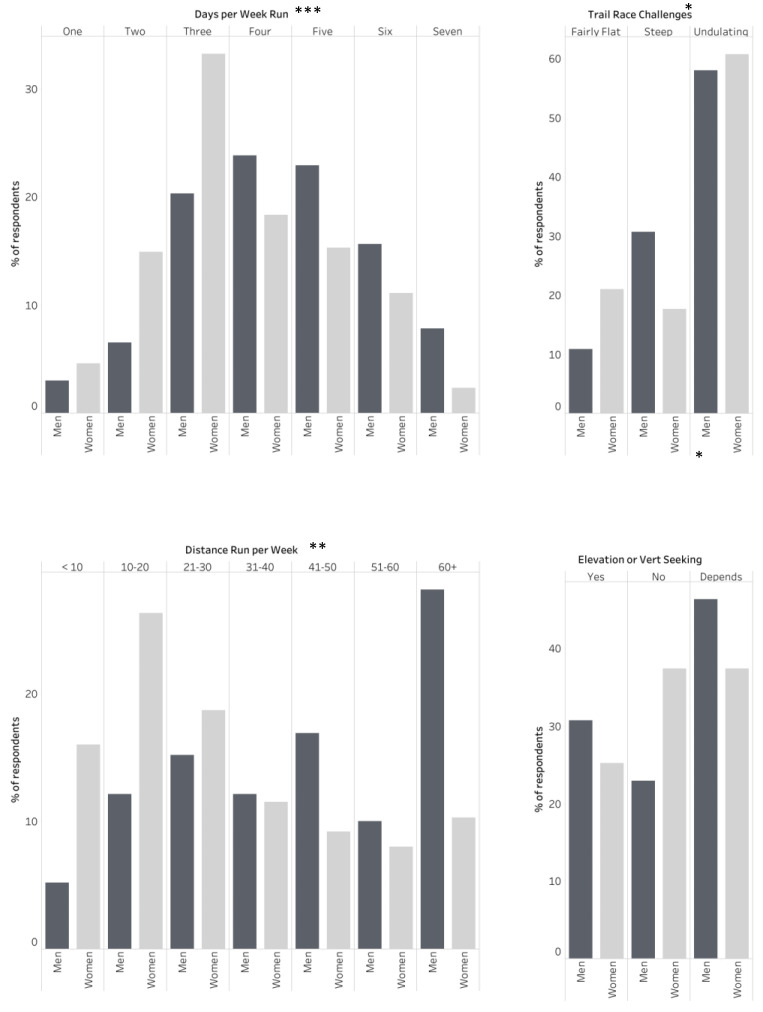
Graphical comparison of runner preferences between men and women. * A significant difference with a “small” effect size as per Cohen’s definition. ** A significant difference with a “medium” effect size as per Cohen’s definition. *** A significant difference with a “large” effect size as per Cohen’s definition.

Respondents ran, on average, 4.0 days/week, with men running more days a week (4.4 days/week) on average than women (3.7 days/week) (Figure 1). There was a significant association between gender and the number of days run per week (X2 (6, N = 492) = 31.256, *p* = 0.000), with a strong effect size (V = 0.252). The percentage of respondents running less than 30 km in a week was 45.1%, with a greater percentage of women running less than 30 km (61.0%) than for men (32.5%) (Figure 1). There was a significant association between gender and distance run per week (X2 (6, N = 492) = 53.904, *p* = 0.000), with a medium effect size (V = 0.331). The person who identified as another gender ran 3 days/week.

### 3.2. Social and Environmental Preferences for Trail Running

Most respondents (35.2%) indicated it was not very important for them to run around others (e.g., a social run with a partner or group) (Table 2) (Table A3). However, among those that did indicate some degree of importance (selecting a 3, 4, or 5 on the sliding scale), women represented a significantly greater percentage (27.5%, 11.5%, and 7.3%, respectively) than men (13.0%, 10.0%, and 1.7%, respectively) (X2 (4, N = 466) = 25.086, *p* = 0.000, with a medium effect size (V = 0.232)). The person who identified as another gender indicated it was somewhat important (i.e., a 3 on the scale) to run around others.

A narrow majority (56%) of those that identified as trail runners preferred to run on buffed out trails compared to rocky, more technical terrain (40.3%). Significantly more women preferred buffed out trails (62.6%) to technical terrain (37.0%), and the same was true for men (58.9% and 48.1%, respectively) (X2 (1 N = 492) = 5.51, *p* = 0.019, with a weak negative relationship (*ϕ* = −0.110)). The person who identified as another gender preferred buffed out trails.

A total of 57.2% of trail runner participants seek out “undulating” trail races, 22.6% seek out steep trail races, and 16.3% seek out flat trail races. Both men and women and the person who identified as another gender preferred undulating trails over steep or flat trails. Significantly more men prefer steeper trails (30.7%) than women (17.6%) (X2 (2, N = 490) = 30.086, *p* = 0.000), with a small effect size (V = 0.086) (Figure 1).

A total of 26. 8% of participants self-identified as an “elevation/vert seeker”, while 29.4% did not. However, for most participants, “being an elevation/vert seeker” is largely dependent on the next race/season (40.3%). Women and men differed significantly in how they self-identified in this regard, with less women (25.2%) identifying as being an elevation or vert seeker than men (30.7%) ((X2 (2, N = 493) = 12.087, *p* = 0.0002), with a small effect size (V = 0.157)) (Figure 1). The person who identified as another gender responded that seeking elevation depended on their next race/season.

### 3.3. Concerns for Trail Running

Out of the ten listed items, the leading concern for respondents while trail running was having a slip or fall (55.1%). Of least concern for all respondents was encountering a cliff or precipice (14.3%).

**Figure 2:**
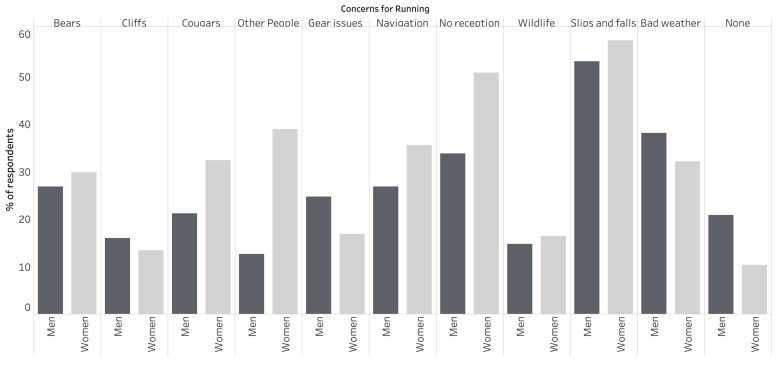
Graphical comparison of runner concerns by gender.

Women respondents reported a much greater fear of people (38.9%) and cougars (32.4%) than men (12.6% and 21.2%, respectively). They were also more concerned with not having cell phone reception in case of emergency while out trail running (50.8%) and navigational challenges (35.5%) than men were (33.8% and 26.8%, respectively). Men reported a greater fear of having gear issues (24.7%) than women (16.8%) and were more likely to respond with “none” (20.8%) for concerns while running than women (10.3%) (Figure 2).

Seventy-seven men and seventy-four women responded to the question about concerns for running with their own responses. Injury, specifically injury while running alone or in a remote area, was the most commonly recurring concern among these responses (41.1%), followed by other wild animals or dogs (13.9%), vehicles (e.g., four-wheelers, cars near trail heads, or on-road sections), or mountain bikes (11.3%), and being harassed or assaulted (9.3%). More men were concerned about injury (51.9%), other wild animals or dogs (15.6%), and vehicles or mountain bikes (13.0%) than women (29.7%, 12.2% and 8.1%, respectively). Conversely, more women were concerned with being harassed or assaulted (16.2%) than men (2.6%).

**Figure 3:**
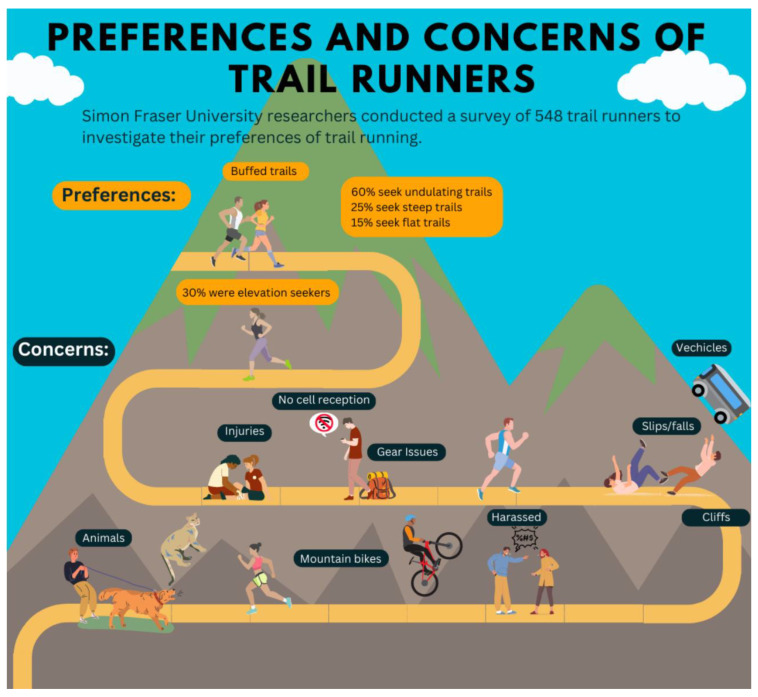
Graphical rendering of principle results of the survey.

The results of the trail runner survey are approximated in Figure 3 which illustrates the main concerns and preferences of trail runners.

## 4. Discussion

This study is the first of its kind to examine the preferences and concerns of recreational trail runners. While preferences vary among trail runners, our results indicate many respondents preferred buffed out trails with a mix of steep and flat sections. Improving cell phone reception in trailed areas, increasing the number and visibility of trail markers, and addressing trail hazards that may cause injury (e.g., removing downed trees, missing planks in board walks, etc.) would alleviate some of the biggest concerns identified by respondents in this study and may make certain environments safer and more appealing for running.

Many of these preferences and concerns have yet to be investigated in the broader runnability literature, making it difficult to place our findings in relation to other studies. We could only find one study that investigated route popularity and slope for recreational road runners in Helsinki, Finland, finding a positive relationship between the two [21]. Far more research has been conducted in the area of walkability with respect to preferences around slope and trail type, though it remains difficult to compare walking to recreational trail running for which objectives may differ widely (e.g., utility vs. recreation) [24,25].

There may be more fruitful comparisons to other sports when examining divergences between genders. In our study, though men and women shared many of the same preferences and concerns regarding trail running environments, some significant differences existed. For example, men reported running 4.4 days per week compared to women’s 3.7 days per week. Similarly, close to 60% of men report running over 30 km on average per week, while less than 40% of women do the same. Similar findings have been reported for other types of recreational physical activity. For example, studies have found more men than women participate in team sports in the USA and Europe [26,27]. However, when it comes to walking for leisure, studies seem to point to the reverse trend, though a systematic review of this literature points to a small effect size, with gender differences diminishing progressively with age [28]. While more research is needed to determine the root of these differences, particularly for running, there have been several studies which suggest that traditional notions of gender norms prioritize recreational activities for men and women within other expected behaviors [29,30,31]. That is, as societal norm dictates that women are expected to provide for the needs of others first, they subsequently neglect their own wants and needs, including leisure participation.

Differences in gender also persisted in concerns respondents had while trail running. More women than men reported a fear of other people while out running. This finding is similar to what was found in road runners and may stem from a fear of being assaulted or harassed, as 12 women reported this fear in the free-text response [23]. It may also be one of the reasons why more women than men reported that it was important for them to run with others and may also be why more woman than men reported to be concerned about not having cell phone reception while out on the trails. Research in this area by Allen-Collinson (2008) supports this thinking, finding that, for women, running with others present could reduce feelings of discomfort and “un-safety” created by negative social interactions such as verbal or physical harassment [22].

Taken together, women’s fears about other people while out running may be limiting the places they run, as well as the frequency and amount they run compared to men. For example, women may choose to run less or for shorter distances if they only feel comfortable running in a certain area or during daylight hours. The desire to run with or around others to mediate or reduce these fears may also hinder the frequency and amount run given the need to co-ordinate schedules with others.

Aside from social and environmental preferences, it is also possible to compare the running profiles of trail versus road runners, based on the study by Schuurman, Rosenkrantz, and Lear (2021) [23]. For example, a greater percentage of trail runners ran 60+ km weeks (16.8%) than road runners (9.1%); however, the number of days per week were similar (4.0 days on average for trail runners and 3.9 days on average for road runners). Other aspects such as the number of years running differ slightly between the studies, with the road runners averaging 10.3 years while the trail runners averaged 11.4 years. Further investigation is warranted to determine whether these differences are significant and what leads to longer participation in trail running compared to road running or vice versa. It may be that trail running has less impact on the body’s joints given the softer running surface of on trails compared to concrete, or that there is greater community aspects in trail running that keep people engaged for longer. However, more research is needed to ascertain the reasons for longevity in the sport and what may extend participation over the lifetime.

Though this research has identified several new findings, there are limitations to the study that require acknowledgement. Respondents from any geography were allowed to participate in the study, allowing for a large sample size and range of responses from both urban and rural areas. While this is an asset to the study, our findings could have been better contextualized had we asked respondents to geographically identify where they typically run and when. This missing information would have allowed us to split the inherent relationships between the data, allowing us to analyze and compare runners of similar geography with each other (e.g., the preferences of trail runners living in urban centers could be distinguished from those living in rural towns). A future study that better accounts for these geographical differences would likely highlight more nuanced preferences and we suggest further investigation along these lines. Our recruitment of runners was also heavily targeted to running groups and magazines based in Canada and the United States, due to the authors’ networks and knowledge of running communities in these areas. It is almost certain that trail runners from these countries are over-represented in our sample, though, as mentioned earlier, geographical context is lacking. This may reduce the generalizability of our study, especially for countries beyond Canada and the United States. It is possible that certain ages and genders are also over or under-represented; however, no literature has defined what representative means for this population. Finally, although we could not conduct statistical analysis to determine if respondents’ preferences and concerns for trail running significantly differed according to age, our descriptive analysis of the data suggests that certain age groups differ from others in this respect. Additional research on these differences is warranted to determine whether and how age groups should be considered in planning trail running routes.

## 5. Conclusions

In summary, this study strengthens our understanding of the recreational trail runner, including their social and environmental preferences and concerns. This information is vital to designing and maintaining environments that encourage trail running, and which promote the many mental and physical health benefits associated with the sport. Continued research into this area is critical to furthering active lifestyles and developing conducive trail running environments for all.

## Figures and Tables

**Table 1:** Comparisons of runner characteristics between men and women.

Variable	Overall Sample n (%)	Men	Women	Chi-Square Tests of
		n (%)	n (%)	Independence
Age in Years				
19–24	115 (22.0)	35 (15.2)	79 (30.2)	*Χ*^2^ (5) = 22.321
25–34	124 (23.7)	60 (26.0)	64 (24.4)	*p* = 0.000
35–44	120 (22.9)	66 (28.6)	54 (20.6)	*V* = 0.213 ***
45–54	86 (16.4)	43 (18.6)	43 (16.4)	*N* = 493
55–64	34 (6.5)	15 (6.5)	19 (7.3)	
65+	15 (2.9)	12 (5.2)	3 (1.1)	
Missing	29 (5.5)	0 (0.0)	0 (0.0)	
Total	523 (100.0)	231 (100.0)	262 (100.0)	
No. of Years Running				
0–5	171 (32.7)	77 (33.3)	93 (35.5)	*Χ*^2^ (6) = 18.143
6–10	139 (26.6)	65 (28.1)	74 (28.2)	*p* = 0.316
11–15	57 (10.9)	25 (10.8)	32 (12.2)	*V* = 0.067
16–20	50 (9.6)	23 (10.0)	27 (10.3)	*N* = 483
21–25	19 (3.6)	9 (3.9)	10 (3.8)	
26–30	23 (4.4)	12 (5.2)	11 (4.2)	
30+	25 (4.8)	13 (5.6)	12 (4.6)	
Missing	39 (7.5)	7 (3.0)	3 (1.1)	
Total	523 (100.0)	231 (100.0)	262 (100.0)	
Distance (Km)/Week				
<10	54 (10.3)	12 (5.2)	42 (16.0)	*Χ*^2^ (6) = 53.904
10–20	98 (18.7)	28 (12.1)	69 (26.3)	*p* = 0.000
21–30	84 (16.1)	35 (15.2)	49 (18.7)	*V* = 0.331 **
31–40	58 (11.1)	28 (12.1)	30 (11.5)	*N* = 492
41–50	63 (12.0)	39 (16.9)	24 (9.2)	
51–60	44 (8.4)	23 (10.0)	21 (8.0)	
60+	92 (17.6)	65 (28.1)	27 (10.3)	
Missing	30 (5.7)	1 (0.4)	0 (0.0)	
Total	523 (100.0)	231 (100.0)	262 (100.0)	
No. of Days Running/Week				
1	19 (3.6)	7 (3.0)	12 (4.6)	*Χ^2^* (6) = 31.256
2	54 (10.3)	15 (6.5)	39 (14.9)	*p* = 0.000
3	135 (25.8)	47 (20.3)	87 (33.2)	*V* = 0.252 ***
4	103 (19.7)	55 (23.8)	48 (18.3)	*N* = 492
5	93 (17.8)	53 (22.9)	40 (15.3)	
6	65 (12.4)	36 (15.6)	29 (11.1)	
7	24 (4.6)	18 (7.8)	6 (2.3)	
Missing	30 (5.7)	0 (0.0)	1 (0.4)	
Total	523 (100.0)	231 (100.0)	262 (100.0)	

*V* = effect size (Cramer’s *V*). ** A significant difference with a “medium” effect size as per Cohen’s definition. *** A significant difference with a “large” effect size as per Cohen’s definition.

**Table 2:** Comparisons of runner preferences and concerns between men and women.

Variable	Overall Sample n (%)	Men	Women	Chi-Square Tests of
		n (%)	n (%)	Independence
Importance of Running around Others				
1 (Not important)	192 (36.7)	98 (42.4)	89 (34.0)	
2	104 (19.9)	57 (24.7)	44 (16.8)	*Χ*^2^ (4) = 25.086
3	108 (20.7)	30 (13.0)	72 (27.5)	*p* = 0.000
4	54 (10.3)	23 (10.0)	30 (11.5)	*V* = 0.232 **
5 (Very important)	26 (5.0)	4 (1.7)	19 (7.3)	*N* = 466
Missing	40 (7.6)	19 (8.2)	8 (3.1)	
Total	523 (100.0)	231 (100.0)	262 (100.0)	
Preferred Running Surface				
Buffed out trail	293 (56.0)	120 (51.9)	164 (62.6)	*Χ*^2^ (1) = 5.5143
Rocky terrain	211 (40.3)	111 (48.1)	97 (37.0)	*p = 0.019*
				*ϕ* = −0.110
Missing	19 (3.6)	0 (0.0)	1 (0.4)	*N* = 492
Total	523 (100.0)	231 (100.0)	262 (100.0)	
Concerns for Running (more than one response permitted)				
Bears	145 (27.7)	62 (26.8)	78 (29.8)	
Fear of people	135 (25.8)	29 (12.6)	102 (38.9)	
Cougars	136 (26.0)	49 (21.2)	85 (32.4)	
Other wildlife	81 (15.5)	34 (14.7)	43 (16.4)	
Gear issues	107 (20.5)	57 (24.7)	44 (16.8)	
No cell reception	222 (42.4)	78 (33.8)	133 (50.8)	*NA*
Slips and falls	288 (55.1)	123 (53.2)	151 (57.6)	*N* = 523
Navigational challenges	162 (31.0)	62 (26.8)	93 (35.5)	
Cliffs or precipices	75 (14.3)	37 (16.0)	35 (13.4)	
Sudden weather/storms	184 (35.2)	88 (38.1)	84 (32.1)	
None	79 (15.1)	48 (20.8)	27 (10.3)	
Other	152 (29.1)	77 (33.3)	74 (28.2)	
What types of trail race challenges do you seek?				
Fairly flat	85 (16.3)	25 (10.8)	55 (21.0)	*Χ*^2^ (2) = 30.086
Steep	118 (22.6)	71 (30.7)	46 (17.6)	*p* = 0.000
Undulating	299 (57.2)	134 (58.0)	159 (60.7)	*V* = 0.086 *
Missing	21 (4.0)	1 (0.4)	2 (0.8)	*N* = 490
Total	523 (100.0)	231 (100.0)	262 (100.0)	
Are you an elevation/vert seeker?				
Yes	140 (26.8)	71 (30.7)	66 (25.2)	*Χ*^2^ (2) = 12.087
No	154 (29.4)	53 (22.9)	98 (37.4)	*p* = 0.002
Depending on next race/season	211 (40.3)	107 (46.3)	98 (37.4)	*V* = 0.157 *
Missing	18 (3.4)	0 (0.0)	0 (0.0)	*N* = 493
Total	523 (100.0)	231 (100.0)	262 (100.0)	

*V* = effect size (Cramer’s *V*). * A significant difference with a “small” effect size as per Cohen’s definition. ** A significant difference with a “medium” effect size as per Cohen’s definition. *ϕ* = Phi coefficient; can range from −1 to 1, with −1 indicating a perfect negative relationship and +1 indicating a perfect positive relationship.

## Data Availability

The data presented in this study are openly available at www.runnerstudy.ca.

## Appendix A

**Table A1:** Questionnaire.

No.	Question
**1**	How important is it for you to run around/with others? (scale from 1 to 5, with 1 being not important and 5 being very important)
**2**	What are your primary concerns for your safety while running? (Check all that apply) Bears; Fear of people; Cougars; Other wildlife; Not having the right gear/malfunctioning gear; Not having cell phone reception in case of emergencies; Slips and falls; Navigation challenges; Cliffs or precipices; Sudden weather changes/storms; None. Other (please specify)
**3**	What is your preferred running surface? Technical trails (photo of a rocky, single-track trail); Buffed out trails (photo of a smooth, wider trail).
**4**	What type of trail races/challenges do you seek? Undulating; Fairly flat; Steep.
**5**	Are you an elevation (“vert”) seeker? Yes; No; Depending on my next race/season.
**6**	How many years have you been running? (single-line text box)
**7**	Approximately how many kilometers do you run a week? Less than 10; 10–20; 20–30; 30–40; 40–50; 50–60; 60+.
**8**	How many days a week do you run? One; Two; Three; Four; Five; Six; Seven.
**9**	What is your gender? Woman; Man; Other.
**10**	What is your age? 19–24; 25–34; 35–44; 45–54; 55–64; 65+.

**Table A2:** Breakdown of trail runner characteristics by age.

Variable	Overall Sample n (%)	19–24 n (%)	25–34 n (%)	35–44 n (%)	45–54 n (%)	55–64 (%)	65+ (%)	Age Not Answered
Gender								
Man	231 (44.2)	35 (30.4)	60 (48.4)	66 (55.0)	43 (50.0)	15 (44.1)	12 (80.0)	0 (0.0)
Woman	262 (50.1)	79 (68.7)	64 (51.6)	54 (45.0)	43 (50.0)	19 (55.9)	3 (20.0)	0 (0.0)
Other	1 (0.2)	1 (0.9)	0 (0.0)	0 (0.0)	0 (0.0)	0 (0.0)	0 (0.0)	0 (0.0)
Missing	29 (5.5)	0 (0.0)	0 (0.0)	0 (0.0)	0 (0.0)	0 (0.0)	0 (0.0)	29 (100.0)
Total	523 (100.0)	115 (100.0)	124 (100.0)	120 (100.0)	86 (100.0)	34 (100.0)	15 (100.0)	29 (100.0)
No. of years running								
0–5	171 (32.7)	64 (55.7)	57 (46.0)	33 (27.5)	14 (16.3)	1 (2.9)	2 (13.3)	0 (0.0)
6–10	139 (26.6)	39 (33.9)	31 (25.0)	38 (31.7)	23 (26.7)	6 (17.6)	2 (13.3)	0 (0.0)
11–15	57 (10.9)	8 (7.0)	21 (16.9)	17 (14.2)	8 (9.3)	3 (8.8)	0 (0.0)	0 (0.0)
16–20	50 (9.6)	2 (1.7)	9 (7.3)	15 (12.5)	14 (16.3)	7 (20.6)	3 (20.0)	0 (0.0)
21–25	19 (3.6)	1 (0.9)	2 (1.6)	7 (5.8)	7 (8.1)	2 (2.9)	0 (0.0)	0 (0.0)
26–30	15 (2.9)	0 (0.0)	1 (0.8)	10 (8.3)	1 (1.2)	3 (8.8)	0 (0.0)	0 (0.0)
30+	33 (6.3)	0 (0.0)	0 (0.0)	0 (0.0)	15 (17.4)	11 (32.4)	7 (46.7)	0 (0.0)
Missing	39 (7.5)	1 (0.9)	3 (2.4)	0 (0.0)	4 (4.7)	1 (2.9)	1 (6.7)	29 (100.0)
Total	523 (100.0)	115 (100.0)	124 (100.0)	120 (100.0)	86 (100.0)	34 (100.0)	15 (100.0)	29 (100.0)
Km distance/week								
<10	54 (10.3)	31 (27.0)	9 (7.3)	7 (5.8)	4 (4.7)	2 (5.9)	1 (6.7)	0 (0.0)
10–20	98 (18.7)	34 (29.6)	25 (20.2)	16 (13.3)	12 (14.0)	8 (23.5)	3 (20.0)	0 (0.0)
21–30	84 (16.1)	22 (19.1)	17 (13.7)	22 (18.3)	17 (19.8)	4 (11.8)	2 (13.3)	0 (0.0)
31–40	58 (11.1)	12 (10.4)	13 (10.5)	7 (5.8)	11 (12.8)	12 (35.3)	3 (20.0)	0 (0.0)
41–50	63 (12.0)	3 (2.6)	17 (13.7)	21 (17.5)	16 (18.6)	4 (11.8)	2 (13.3)	0 (0.0)
50–60	44 (8.4)	5 (4.3)	14 (11.3)	15 (12.5)	7 (8.1)	2 (5.9)	1 (6.7)	0 (0.0)
60+	92 (17.6)	7 (6.1)	29 (23.4)	32 (26.7)	19 (22.1)	2 (5.9)	3 (20.0)	0 (0.0)
Missing	30 (5.7)	1 (0.9)	0 (0.0)	0 (0.0)	0 (0.0)	0 (0.0)	0 (0.0)	29 (100.0)
Total	523 (100.0)	115 (100.0)	124 (100.0)	120 (100.0)	86 (100.0)	34 (100.0)	15 (100.0)	29 (100.0)
No. of Days Running/Week								
1	19 (3.6)	7 (6.1)	6 (4.8)	4 (3.3)	1 (1.2)	0 (0.0)	1 (6.7)	0 (0.0)
2	54 (10.3)	21 (18.3)	16 (12.9)	6 (18.3)	6 (7.0)	3 (8.8)	2 (13.3)	0 (0.0)
3	135 (25.8)	35 (30.4)	30 (24.2)	32 (26.7)	23 (26.7)	13 (38.2)	2 (13.3)	0 (0.0)
4	103 (19.7)	15 (13.0)	23 (18.5)	25 (20.8)	25 (29.1)	12 (35.3)	3 (20.0)	0 (0.0)
5	93 (17.8)	17 (14.8)	25 (20.2)	26 (21.7)	17 (19.8)	5 (14.7)	3 (20.0)	0 (0.0)
6	65 (12.4)	15 (13.0)	13 (10.5)	22 (18.3)	12 (14.0)	1 (2.9)	2 (13.3)	0 (0.0)
7	24 (4.6)	5 (4.3)	10 (8.1)	5 (21.7)	2 (2.3)	0 (0.0)	2 (13.3)	0 (0.0)
Missing	30 (5.7)	0 (0.0)	1 (4.8)	0 (0.0)	0 (0.0)	0 (0.0)	0 (0.0)	29 (100.0)
Total	523 (100.0)	115 (100.0)	124 (100.0)	120 (100.0)	86 (100.0)	34 (100.0)	15 (100.0)	29 (100.0)

**Table A3:** Breakdown of trail runner preferences and concerns by age.

Variable	Overall Sample n (%)	19–24 n (%)	25–34 n (%)	35–44 n (%)	45–54 n (%)	55–64 (%)	65+ (%)	Age Not Answered
Importance of running around Others								
1 (not important)	192 (36.7)	40 (34.8)	38 (30.6)	49 (40.8)	39 (45.3)	16 (47.1)	5 (33.3)	5 (17.2)
2	104 (19.9)	23 (20.0)	28 (22.6)	27 (22.5)	17 (19.8)	5 (14.7)	1 (6.7)	3 (10.3)
3	108 (20.7)	28 (24.3)	34 (27.4)	16 (13.3)	16 (18.6)	3 (8.8)	6 (40.0)	5 (17.2)
4	54 (10.3)	12 (10.4)	14 (11.2)	12 (10.0)	5 (5.8)	8 (23.5)	2 (13.3)	1 (3.4)
5 (very important)	25 (4.8)	7 (6.1)	5 (4.0)	5 (4.2)	5 (5.8)	1 (2.9)	0 (0.0)	2 (6.8)
Missing	40 (7.6)	5 (4.3)	5 (4.0)	11 (9.2)	4 (4.7)	1 (2.9)	1 (6.7)	13 (44.8)
Total	523 (100.0)	115 (100.0)	124 (100.0)	120 (100.0)	86 (100.0)	34 (100.0)	15 (100.0)	29 (100.0)
Concerns for safety while running (>1 response allowed)								
Bears	158 (9.1)	29 (7.6)	45 (10.6)	37 (8.6)	20 (7.8)	9 (7.7)	14 (24.1)	4 (5.8)
Fear of people	135 (7.8)	34 (8.9)	32 (7.5)	33 (7.7)	22 (8.6)	7 (6.0)	3 (5.2)	4 (5.8)
Cougars	136 (7.8)	26 (6.8)	42 (9.9)	36 (8.4)	17 (6.6)	9 (7.7)	4 (6.9)	2 (2.9)
Other wildlife	81 (4.7)	22 (5.8)	16 (3.8)	21 (4.9)	13 (5.1)	3 (2.6)	2 (3.4)	4 (5.8)
Gear issues	107 (6.2)	28 (7.3)	28 (6.6)	23 (5.3)	14 (5.4)	7 (6.0)	1 (1.7)	6 (8.7)
No cell reception	222 (12.8)	57 (15.0)	53 (12.4)	52 (12.1)	29 (11.3)	16 (13.7)	5 (8.6)	10 (14.5)
Slips and falls	288 (16.6)	56 (14.7)	66 (15.5)	67 (15.5)	51 (19.8)	26 (22.2)	9 (15.5)	13 (18.8)
Navigation issues	162 (9.3)	37 (9.7)	37 (10.0)	43 (10.0)	21 (8.2)	14 (12.0)	3 (5.2)	7 (10.1)
Cliffs or precipices	75 (4.3)	12 (3.1)	17 (5.1)	22 (5.1)	12 (4.7)	5 (4.3)	4 (6.9)	3 (4.3)
Sudden weather	184 (10.6)	46 (12.1)	48 (9.3)	40 (9.3)	26 (10.1)	8 (6.8)	5 (8.6)	11 (15.9)
None	79 (4.5)	17 (4.5)	16 (4.2)	18 (4.2)	12 (4.7)	5 (4.3)	7 (12.1)	4 (5.8)
Other	112 (6.4)	17 (4.5)	26 (9.0)	39 (9.0)	20 (7.8)	8 (6.8)	1 (1.7)	1 (1.4)
Total	1739 (100.0)	381 (100.0)	426 (100.0)	431 (100.0)	257 (100.0)	117 (100.0)	58 (100.0)	69 (100.0)
Preferred running surface								
Buffed out trails	293 (56.0)	97 (84.3)	62 (50.0)	50 (41.7)	45 (52.3)	21 (61.8)	10 (66.7)	8 (27.6)
Technical trails	211 (40.3)	18 (15.7)	62 (50.0)	70 (58.3)	41 (47.7)	12 (35.3)	5 (33.3)	3 (10.3)
Missing	19 (3.6)	0 (0.0)	0 (0.0)	0 (0.0)	0 (0.0)	1 (2.9)	0 (0.0)	18 (62.1)
Total	523 (100.0)	115 (100.0)	124 (100.0)	120 (100.0)	86 (100.0)	34 (100.0)	15 (100.0)	29 (100.0)
Prefers elevation								
Yes	140 (26.8)	24 (20.9)	43 (34.7)	35 (29.2)	24 (27.9)	8 (23.5)	3 (20.0)	3 (5.6)
No	154 (29.4)	46 (40.0)	33 (26.6)	29 (24.2)	22 (25.6)	14 (41.2)	7 (46.7)	3 (5.6)
Depending on next race/season	211 (40.3)	45 (39.1)	48 (38.7)	56 (46.7)	40 (46.5)	12 (35.3)	5 (33.3)	5 (9.3)
Missing	18 (3.4)	0 (0.0)	0 (0.0)	0 (0.0)	0 (0.0)	0 (0.0)	0 (0.0)	18 (79.6)
Total	523 (100.0)	115 (100.0)	124 (100.0)	120 (100.0)	86 (100.0)	34 (100.0)	15 (100.0)	29 (100.0)
Preferred trail race challenges								
Undulating	299 (57.2)	62 (53.9)	67 (54.0)	72 (60.0)	55 (64.0)	26 (76.5)	11 (73.3)	6 (20.7)
Fairly flat	85 (16.3)	39 (33.9)	17 (13.7)	9 (7.5)	8 (9.3)	4 (11.8)	4 (26.7)	4 (13.8)
Steep	118 (22.6)	13 (11.3)	40 (32.3)	38 (31.7)	23 (26.7)	3 (8.8)	0 (0.0)	1 (3.4)
Missing	21 (4.0)	1 (0.9)	0 (0.0)	1 (0.8)	0 (0.0)	1 (2.9)	0 (0.0)	18 (62.1)
Total	523 (100.0)	115 (100.0)	124 (100.0)	120 (100.0)	86 (100.0)	34 (100.0)	15 (100.0)	29 (100.0)
